# Measuring sleep health in primary school-aged children: A systematic review of instruments and their content validity

**DOI:** 10.1093/sleep/zsac215

**Published:** 2022-09-10

**Authors:** Maj-Britt M R Inhulsen, Maartje M van Stralen, Caroline B Terwee, Joanne K Ujcic-Voortman, Jacob C Seidell, Vincent Busch

**Affiliations:** Department of Health Sciences, Faculty of Science, Vrije Universiteit Amsterdam, Amsterdam Public Health Research Institute, Amsterdam, the Netherlands; Sarphati Amsterdam, Public Health Service (GGD), City of Amsterdam, Amsterdam, the Netherlands; Department of Health Sciences, Faculty of Science, Vrije Universiteit Amsterdam, Amsterdam Public Health Research Institute, Amsterdam, the Netherlands; Department of Epidemiology and Data Science, Amsterdam UMC, Vrije Universiteit Amsterdam, Amsterdam Public Health Research Institute, Amsterdam, the Netherlands; Sarphati Amsterdam, Public Health Service (GGD), City of Amsterdam, Amsterdam, the Netherlands; Department of Health Sciences, Faculty of Science, Vrije Universiteit Amsterdam, Amsterdam Public Health Research Institute, Amsterdam, the Netherlands; Sarphati Amsterdam, Public Health Service (GGD), City of Amsterdam, Amsterdam, the Netherlands; Sarphati Amsterdam, Public Health Service (GGD), City of Amsterdam, Amsterdam, the Netherlands

**Keywords:** children, COSMIN, measurement properties, questionnaires, sleep

## Abstract

**Study Objectives:**

This review aimed to summarize instruments that measure one or more domains of sleep health (i.e. duration, quality, efficiency, timing, daytime sleepiness and sleep-related behaviors) in a general population of 4–12-year old children, and to assess these instruments’ content validity. Other measurement properties were evaluated for instruments with indications of sufficient content validity.

**Methods:**

A systematic literature search was performed in PubMed, PsycINFO, Web of Science, and EmBase. Methodological quality, content validity, and other measurement properties were assessed via the COnsensus-based Standards for the selection of health Measurement INstruments (COSMIN) methodology. Instruments with indications of sufficient content validity (i.e. relevance, comprehensiveness and comprehensibility) were further evaluated on other measurement properties (i.e. other aspects of validity, reliability, responsiveness). A modified GRADE approach was applied to determine the quality of evidence.

**Results:**

Twenty instruments, containing 36 subscales, were included. None of the instruments measured all sleep health domains. For five (subscales of) instruments sufficient relevance and comprehensibility was found. The quality of evidence ranged from very low to moderate. For these five instruments all additional measurement properties were assessed. Sufficient results were found for structural validity (*n* = 1), internal consistency (*n* = 1), and construct validity (*n* = 1), with quality of evidence ranging from very low to high.

**Conclusions:**

Several (subscales of) instruments measuring domains of child sleep health showed good promise, demonstrating sufficient relevance, comprehensibility, and some also sufficient results on other measurement properties. However, more high quality studies on instrument development and the evaluation of measurement properties are required.

PROSPERO registration number: CRD42021224109

## Introduction

Healthy sleep is essential for the health and well-being of children. Many studies have shown that chronic insufficient sleep, poor sleep quality and irregular sleep routines in primary school-aged children are risk factors for impaired cognition [1–3], poor academic performance [4, 5], developing behavioral difficulties (e.g. aggression, emotion regulation difficulties [6]), psychosocial problems and obesity [7–9]. Good sleep health is a multidimensional construct that includes several aspects such as proper sleep duration, sleep quality, sleep efficiency (i.e. sleep latency, wake after sleep onset), sleep timing, and the absence of daytime sleepiness [10]. Recently, the definition on what constitutes good sleep health was adapted to pediatrics and was extended with sleep-related behaviors such as bedtime routine consistency [11].

Given the importance for health and well-being of children, stimulating sleep health deserves a prominent place in public health. It is therefore crucial to validly and reliably monitor population sleep health and to evaluate the effectiveness of interventions aimed at improving sleep health [12, 13]. Important in determining the quality of such an instrument for these purposes are its measurement properties. Validity is one of these measurement properties and refers to the extent to which an instrument accurately measures what it intends to measure [14]. Of the domains of validity that can be distinguished, content validity is considered a vital element [15] since it refers to “the degree to which the content of an instrument is an adequate reflection of the construct to be measured” [14]. This measurement property is primarily evaluated with input from the target population as it comprises the relevance, comprehensiveness, and comprehensibility of an instrument. When these aspects of an instrument are insufficient it affects the other measurement properties, which emphasizes the importance of good content validity before assessing other measurement properties.

Aside from validity and other important measurement properties such as reliability and responsiveness, instruments for measuring child sleep health should be feasible to use. Despite being viewed as the gold standard to measure sleep-wake function in clinical settings, polysomnography is not feasible for large epidemiological studies or population-level monitoring due to its costs and the participant burden. Also, polysomnography provides no information on subjective sleep domains like sleep quality and daytime sleepiness (i.e. lacking instrument validity). Furthermore, actigraphy, another validated and often-used measurement method for estimating sleep in children [16–18], is less feasible for population-level use due to its costs and the participant burden. Moreover, actigraphy also does not provide sufficient information on the subjective experiences of sleep and sleepiness, thereby providing only partial data on the full concept of child sleep health (i.e. lacking instrument validity). Therefore, objective methods like polysomnography and actigraphy serve a clear purpose in identifying whether an individual meets a diagnostic criterion for a sleep disorder or disturbance such as insomnia, but are not suitable to measure the full concept of sleep health in children through large population level monitoring.

Subjective child- or parent-report measures of sleep health are expected to be the most suitable for use in large population based studies, given the practical limitations of polysomnography and actigraphy. A wide range of subjective instruments are available, yet the current literature lacks a systematic appraisal of the quality and measurement properties of these available instruments, complicating the choice for an adequate instrument in a particular context [19, 20]. Previous reviews on sleep measures did not cover all core domains of sleep health [21], or did not systematically review the evidence of measurement properties [19, 20, 22], or did not focus on primary school-aged children (i.e. 4–12 years) specifically [23], and/or did not evaluate the measurement property of content validity.

Therefore, the current study aimed to present an overview of all child- or parent-reported instruments that can be used to assess one or more of the elements of the current definition of child sleep health in a general population of children aged 4–12 years, and that were validated to at least some extent. We performed a comprehensive assessment of their content validity. Only those with indications of adequate content validity were further assessed on other measurement properties (i.e. other aspects of validity, reliability, responsiveness). The purpose was to provide recommendations for instruments that are suitable for population-level monitoring of child sleep health for evaluative purposes.

## Methods

For this study the COnsensus-based Standards for the selection of health Measurement INstruments (COSMIN) guideline for systematic reviews of Patient-Reported Outcome Measures (PROMs), including methodology for assessing content validity, was used [15, 24]. The Preferred Reporting Items for Systematic Reviews and Meta-Analyses (PRISMA) reporting guidelines were followed. This review was registered at PROSPERO, the international prospective register of systematic reviews (CRD42021224109).

### Literature search and eligibility criteria

This systematic literature review was designed based on the University of York’s Centre for Reviews and Dissemination (CRD) handbook Systematic Reviews: CRD’s Guidance for Undertaking Reviews in Health Care [25]. The databases PubMed, PsycInfo, Web of Science and EmBase were systematically searched until August 2021. In addition, literature reviews and grey literature (e.g. reports, dissertations, manuals) were screened for additional studies and/or instruments. For the search, the search filter from Terwee at al. was used for identifying studies on the measurement properties of sleep measurement instruments [26]. The following criteria were used for inclusion of studies: first, the instrument measured an aspect of the (pediatric) sleep health definition, including: (1) sleep duration, (2) sleep quality, (3) sleep efficiency, (4) timing, (5) daytime sleepiness, and (6) sleep-related behaviors. Second, the instrument was developed for children with an average age between 4 and 12 years old (<13 years) from the general population instead of a specific subgroup (e.g. focused on a clinical sleep disorder). Third, the instrument was used for evaluative purposes and was either child- or parent-reported. Fourth, the study aimed to evaluate one or more measurement properties. Fifth, the study entailed original empirical research published in full-text in peer reviewed scientific journals. Sixth, the study was published in English or Dutch. No studies were excluded based on publication date. For more details on the search strategies in all four databases, see Appendix 1.

### Paper selection procedures

Three independent reviewers (MI, VB, MvS) performed the title-abstract selection. The selection of full-text papers, methodological quality assessment and the assessment of the measurement properties of the studied instruments were independently reviewed by two reviewers (MI and VB). All papers selected for full-text review were also used for secondary searches via backward tracking (reference tracking) and forward tracking, i.e. checking papers that cited the included papers. Additionally, all included papers were searched for references of studies or manuals that reported on the development or assessment of measurement properties.

If a paper was included via these secondary searches it underwent the same selection process as the papers included by the original search. Any disagreement between the two reviewers was resolved with a third reviewer (MvS). If the full text version of a paper could not be retrieved, its corresponding authors were contacted and asked for the full text version. If we did not receive the full text version after multiple efforts, we continued without that article (n=5).

### Data extraction

The following data were extracted regarding characteristics of the included instruments: target population (i.e. age of the population for which the instrument was developed), number of items, child- or parent reported. Additional data that were extracted: study population (i.e. population included in the study), time interval (for test-retest reliability studies), comparison measure (for construct validity studies), and the results of the examined measurement properties (i.e. validity, reliability, responsiveness).

### Methodological quality assessment

The methodological quality assessment was performed using the COSMIN Risk of Bias checklist [24]. This checklist provides methodological standards for each measurement property to assess the risk of bias (i.e. trustworthiness of results). Each standard was scored by two reviewers (MI and VB) independently on a 4-point rating scale, ranging from “very good”, “adequate”, “doubtful”, to “inadequate”. A total score was then determined via a “worst score counts” method in which the worst score on a particular domain determined the final methodological quality.

### Rating of study results

All studies on measurement properties were rated against the criteria for good measurement properties [27]. These criteria indicate for each measurement property which outcomes are considered sufficient (+); insufficient (−); inconsistent (±); or indeterminate (?). Criteria for content validity and other aspects of validity, reliability, and responsiveness can be found below.

### Evaluation of content validity

Content validity assessment consisted of 1) the evaluation of the methodological quality of the instrument’s development study, and 2) the evaluation of the methodological quality of the available content validity studies. First, for the methodological quality assessment of the instrument’s development study, the COSMIN standards comprise items on the concept elicitation study performed with the target population to identify relevant and comprehensive items for the new instrument. The second part consists of items on the interview study (or other pilot test) performed with the target population to evaluate comprehensibility and comprehensiveness of the instrument. Second, to assess the methodological quality of the studies on content validity, the COSMIN standards comprise items on the instrument’s relevance, comprehensiveness and comprehensibility from the user’s perspective, as well as the relevance and comprehensiveness from the professional’s perspective [15].

Only instruments with indications of sufficient content validity (i.e. sufficient results on either relevance, comprehensiveness or comprehensibility, regardless of the level of evidence) were further evaluated on other measurement properties (i.e. validity, reliability, responsiveness). Since the current review aims to provide an overview of instruments with indications of sufficient content validity, we slightly diverged from the COSMIN manual, which only excludes instruments with high quality evidence of inadequate content validity [24].

Before content validity was assessed, the measured construct(s) of the instrument were classified according to the sleep health definitions of Buysse and Meltzer [10, 11]. For multidimensional instruments, i.e. instruments that consist of multiple subscales, each subscale was classified according to the same definitions. Therefore the relevance and comprehensiveness of an instrument (and its subscales) were rated according to these definitions.

### Evidence synthesis of content validity

Based on a summary of the evidence of the previous two steps, the content validity of the instrument was rated based on 10 criteria of good content validity [15]. In addition, the content of the instrument itself was rated by two reviewers (MI and VB). This aspect included whether the reviewers perceived the instruments as relevant, comprehensive and comprehensible. In the next step an overall score for the content validity of each instrument was determined by qualitatively summarizing the evidence using the COSMIN guidelines (i.e. results of PROM development and available content validity studies) and the reviewers rating. The reviewer rating was also separately reported, thereby slightly deviating from the COSMIN manual. The overall score for each instrument was based on the totality of the scores for the concepts relevance, comprehensiveness and comprehensibility, and it provides a classification of sufficient (+), insufficient (−), inconsistent (±), or indeterminate (?).

### Evaluation of internal structure: structural validity, internal consistency, and cross-cultural validity

Internal structure refers to the relatedness between items of a scale or subscale. To assess the internal structure three measurement properties should be evaluated: 1) structural validity, 2) internal consistency, and 3) cross-cultural validity. Both structural validity and internal consistency can only be assessed when the instrument is based on a reflective model, i.e. wherein the items are manifestations of the same underlying construct and thus are expected to be correlated [27].

Structural validity is “the degree to which the scores of a questionnaire are an adequate reflection of the dimensionality of the construct to be measured” [14] and is usually evaluated by factor analysis. Exploratory factor analysis were considered sufficient when the first factor accounted for ≥20% of the variability and when the ratio of the variance explained by the first factor divided by the second factor was >4 [28], whereas confirmatory analysis were considered sufficient when the comparative fit index or Tucker-Lewis index was >0.95, the mean square error of approximation was <0.06, or the standardized root mean residual was <0.08 [27].

Internal consistency is “the degree of the inter-relatedness among items” [14] and can be evaluated by Cronbach’s alpha. Cronbach’s alpha values of ≥0.70 for each unidimensional scale or subscale and at least low quality of evidence for sufficient structural validity were considered sufficient [27].

As none of the studies evaluated cross-cultural validity, these criteria are not reported.

### Evaluation of remaining measurement properties: reliability, measurement error, criterion validity, construct validity, and responsiveness

Reliability is “the degree to which the measurement is free from measurement error” [14]. Reliability was considered sufficient by either intraclass correlation coefficients (ICC) or Kappa (K) values of ≥0.70 [27], or by Pearson or Spearman correlations of ≥0.80 [29].

Construct validity is “the degree to which the scores of an instrument are consistent with hypotheses” (e.g. with regards to internal relationships, relationships to scores of other instruments, or differences between groups) [14]. For instruments measuring a similar construct we considered correlations ≥0.50 as sufficient. For instruments measuring related constructs we considered 0.30–0.50 as sufficient and <0.30 for instruments measuring unrelated constructs [27].

As none of the included studies evaluated measurement error, criterion validity, and responsiveness, these criteria are not reported.

### Grading the quality of evidence

By using the Grading of Recommendations Assessment, Development, and Evaluation (GRADE) approach, a grading for the quality of the evidence (i.e. high, moderate, low, or very low quality of evidence) was determined as the last step [27]. The grading was based on four factors: (1) risk of bias (i.e. the methodological quality of the studies), (2) inconsistency (i.e. unexplained inconsistency of results across studies), (3) imprecision (i.e. total sample size of the available studies, and (4) indirectness (i.e. evidence from different populations than the population of interest in this review). Based on the presence of these four factors, the quality of evidence was subsequently downgraded, starting from high quality, by one, two or three levels per factor [27].

## Results

**Figure 1.**
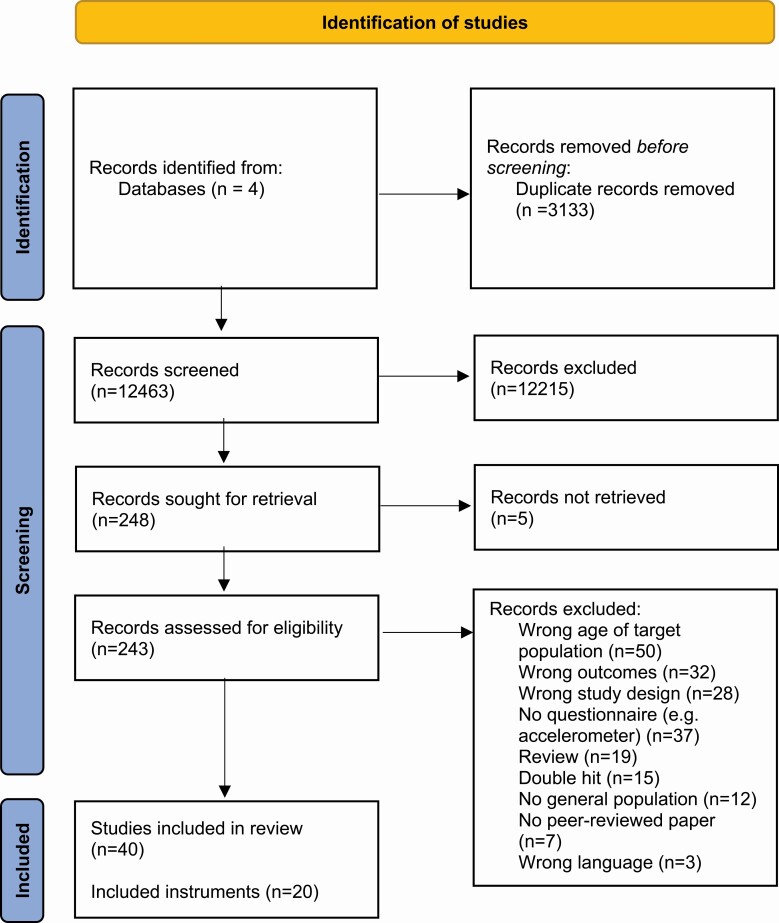
PRISMA flow diagram.

Systematic literature searches yielded 12 463 articles after removal of duplicates. Articles were screened on title and abstract after which the large majority of articles were excluded because they did not meet the inclusion criteria, with 248 articles remaining for full review of which five could not be retrieved. One article was found through backward tracking. Following full review, an additional 203 articles were excluded, mainly because they did not include the right age range of the study population (*n* = 50), the study did not concern a questionnaire (*n* = 37), they did not include the right outcome (i.e. domains of sleep health) (*n* = 32) or because the study did not assessed measurement properties (*n* = 28). After full review 40 articles remained for further assessment. See figure 1 for the PRISMA flowchart for details.

### Instrument characteristics

The 40 included articles comprised studies on 20 instruments, containing, in total, 36 subscales measuring one or more domains of sleep health. Table 1 presents an overview of the 20 instruments and describes the age-range of the target population, the number of items (per subscale) and whether the instrument is parent- and/or child-reported. It also provides an overview of which sleep health domains are measured per instrument and subscale. Most instruments were meant for children from the age of eight onwards and included instruments ranged from five to 60 items. Of the 20 instruments 10 were child-reported, seven were parent-reported, two combined questions for parents and children and one was child-reported but could be reported by parents. Most instruments were multidimensional as they measured two or more domains of sleep health. We found 10 (subscales of) instruments measuring sleep duration, 5 (subscales of) instruments measuring sleep quality, 13 (subscales of) instruments measuring sleep efficiency, 6 (subscales of) instruments measuring sleep timing, 12 (subscales of) instruments measuring daytime sleepiness and 13 (subscales of) instruments measuring sleep-related behaviors. Some (subscales of) instruments measured multiple domains within one (sub)scale. Five instruments measured sleep in addition to other health-related behaviors.

**Table 1.** Characteristics of the included instruments measuring domains of sleep health in children aged 4–12 years

Sleep health domains measured									
Instrument (and subscales)	Target population	Number of items	Reporter	Sleep duration	Sleep quality	Sleep efficiency	Timing	Daytime sleepiness	Sleep-related behaviors
**Bedtime Routines Questionnaire (BRQ) [30]**	2–8	31	Parent						**✓**
**Children’s Report of Sleep Patterns (CRSP) [31]**	8–12	60	Child						
* Sleep patterns*		20		**✓**	**✓**	**✓**	**✓**		
* Sleep hygiene index*		18							**✓**
* Sleep disturbances*		22							
**Children’s Report of Sleep Patterns – sleepiness scale (CRSP-S) [32]**	8–12	5	Child					**✓**	
**Children’s Sleep Behavior Scale (CSBS) [33]**	6–12	22	Parent			**✓**			
**Children’s Sleep Habits Questionnaire (CSHQ) [34]**	4–10	35	Parent						
* Bedtime resistance*		6							**✓**
* Sleep onset delay*		1				**✓**			
* Sleep duration*		3		**✓**					
* Sleep anxiety*		4							
* Night wakings*		3							
* Parasomnias*		7							
* Sleep-disordered breathing*		3							
* Daytime sleepiness*		8						**✓**	
***“CSHQ-short Japan” (CSHQ-s)* [35]**	6–12	19	Parent						
* Bedtime behavior*		4							
* Sleep behavior*		9							
* Difficulty with morning waking*		5						**✓**	
* Hypersomniac symptoms*		1							
**Children’s Sleep Wake Scale (CSWS) [36]**	2–8	25	Parent						
* Going to bed*		5							**✓**
* Falling asleep*		5				**✓**			
* Maintaining sleep*		5							
* Reinitiating sleep*		5				**✓**			
* Returning to wakefulness*		5						**✓**	
**Children’s Sleep Assessment Questionnaire (CSAQ) [37]**	8–12	37 (child) + 6 (parent)	Child and/or parent						
* Sleep hygiene*		16							**✓**
* Sleep quality*		15		**✓**		**✓**	**✓**	**✓**	
* Sleep disturbances*		6							
**Health Behaviour in School-aged Children (HBSC) survey [38]**	11–15	4 (102 items in total)	Child	**✓**					
**Japan Children’s Study Sleep Questionnaire (JCSSQ) [39]**	6–12	Sleep log	Parent	**✓**		**✓**			
**Japanese Sleep Questionnaire for Elementary Schoolers (JSQ-ES) [40]**	6–12	36	Parent						
* Restless legs syndrome*		6							
* Sleep-disordered breathing*		5							
* Morning symptoms*		3							
* Nighttime awakenings*		5							
* Insomnia*		3							
* Excessive daytime sleepiness*		4						**✓**	
* Daytime behavior*		4							
* Sleep habits*		2							
* Irregular/delayed sleep phase*		4							**✓**
**MyDailyMoves (MDM) [41]**	9–12	Timeline format	Child	**✓**	**✓**	**✓**		**✓**	**✓**
**Pediatric Daytime Sleepiness Scale (PDSS) [42]**	11–15	8	Child					**✓**	
**Pediatric Sleep Practices Questionnaire (PSPQ) [43]**	8–17	15	Child						
* Sleep timing*		6		**✓**			**✓**		
* Sleep routines and consistency*		1							**✓**
* Technology use before bedtime*		3							**✓**
* Sleep environment*		4							**✓**
* Need for parental presence*		1							
**Pictorial Sleepiness Scale (PSS) [44]**	>4	7	Child					**✓**	
**PROMIS Pediatric Sleep Health Items (PROMIS-PSHI) [45]**	5-17/8-17	43 (child); 6 (parent)	Child (≥ 8 years) or parent (5-8 years)						
* Sleep onset*		9				**✓**			
* Sleep continuity*		5				**✓**			
* Sleep quality*		8			**✓**				
* Dreams*		2							
* Breathing*		4							
* Parasomnias*		3							
* Daytime sleepiness*		4						**✓**	
* Energy*		2							
* Sleep offset*		3			**✓**				
* Impact- cognitive*		1							
* Impact- activities*		4							
* Impact- affect or behaviors*		4							
**“Simple Self-Report SlSeep Questionnaire” (SSRSQ) [46]**	9–12	4	Child	**✓**					
**Sleep and Lifestyle Questionnaire (SLQ) [47]**	6–16	11	Child (≥ 10 years) or parent (6-9 years)	**✓**		**✓**	**✓**	**✓**	**✓**
**Sleep Self Report (SSR) [48, 49]**	7–12	26	Child		**✓**	**✓**	**✓**	**✓**	**✓**
**Sleep Timing Questionnaire (STQ) [50, 51]**	11–16	18	Child	**✓**		**✓**	**✓**		**✓**

### Content validity

- Quality of development studies

Appendix 2 presents the ratings of the development studies. Only 9 out of 20 instruments were developed in a sample of children and/or parents, but only two studies used qualitative methods for this. For both the Pediatric Sleep Practices Questionnaire and the PROMIS Pediatric Sleep Health Items parents and children were involved in the concept elicitation and this part of the development was therefore considered adequate and very good. Four instruments were pilot tested. Total instrument development was rated inadequate for 17 out of 19 instruments. One instrument was rated as doubtful (MyDailyMoves) and for only two instruments the development was considered adequate (PROMIS Pediatric Sleep Health Items) or very good (Pediatric Sleep Practices Questionnaire).

- *Quality of content validity studies*

Details of the content validity studies can be found in Appendix 3. Of the 40 included articles on 20 instruments only two studied content validity: the Pediatric Sleep Practices Questionnaire and the PROMIS Pediatric Sleep Health Items. Both studies evaluated comprehensibility of the instrument as part of content validity and were of doubtful quality.

- *Evidence synthesis of content validity*

None of the (subscales of) instruments demonstrated sufficient content validity regarding all aspects of content validity (see Table 2). The quality of evidence ranged from very low to moderate. Sufficient relevance was found for the MyDailyMoves (MDM) instrument and the Pictorial Sleepiness Scale (PSS), with moderate and very low quality of evidence, respectively. No sufficient comprehensiveness was found for any (subscales of) instruments. Sufficient comprehensibility was found for MDM and for all subscales of the Pediatric Sleep Practices Questionnaire (PSPQ) and the PROMIS Pediatric Sleep Health Items (PROMIS-PSHI). The quality of evidence was moderate. Sufficient comprehensibility was also found for all subscales of the Children’s Sleep Wake Scale (CSWS) and the Pictorial Sleepiness Scale (PSS), with low and very low quality of evidence, respectively.

**Table 2.** Evidence synthesis on the content validity of instruments measuring domains of sleep health in children aged 4–12 years

	Content validity					
	Relevance		Comprehensiveness		Comprehensibility	
Instrument (and subscales)	Rating of results	Quality of evidence	Rating of results	Quality of evidence	Rating of results	Quality of evidence
**Bedtime Routines Questionnaire (BRQ) [30]**	±	Very low	±	Very low	±	Very low
**Children’s Report of Sleep Patterns (CRSP) [31]**						
* Sleep patterns –* * domain: sleep duration*	±	Very low	±	Very low	±	Very low
* Sleep patterns – domain: sleep quality*	±	Very low	±	Very low	±	Very low
* Sleep patterns – domain: sleep efficiency*	±	Very low	±	Very low	±	Very low
* Sleep patterns - timing*	±	Very low	±	Very low	±	Very low
* Sleep hygiene index*	±	Very low	±	Very low	±	Very low
**Children’s Report of Sleep Patterns – sleepiness scale (CRSP-S**) **[32]**	±	Very low	±	Very low	±	Very low
**Children’s Sleep Behavior Scale (CSBS) [33]**	±	Very low	-	Very low	-	Very low
**Children’s Sleep Habits Questionnaire (CSHQ) [34]**						
* Bedtime resistance – domain: behaviors*	±	Very low	-	Very low	±	Very low
* Sleep onset delay*	±	Very low	-	Very low	±	Very low
* Sleep duration*	±	Very low	±	Very low	±	Very low
* Daytime sleepiness*	±	Very low	±	Very low	±	Very low
***“CSHQ-short Japan” (CSHQ-s)* [35]**						
* Difficulty with morning waking*	±	Very low	-	Very low	±	Very low
**Children’s Sleep Wake Scale (CSWS) [36]**						
* Going to bed*	±	Low	±	Low	+	Low
* Falling asleep*	±	Low	±	Low	+	Low
* Reinitiating sleep*	±	Low	±	Low	+	Low
* Returning to wakefulness*	±	Low	±	Low	+	Low
**Children’s Sleep Assessment Questionnaire (CSAQ) [37]**						
* Sleep hygiene*	±	Very low	-	Very low	±	Very low
* Sleep quality - duration*	±	Very low	-	Very low	±	Very low
* Sleep quality - efficiency*	±	Very low	-	Very low	±	Very low
* Sleep quality –* * timing*	±	Very low	-	Very low	±	Very low
* Sleep quality – daytime sleepiness*	±	Very low	-	Very low	±	Very low
Health Behaviour in School-aged Children (HBSC) survey^*^ [38]						
* Sleep subscale – domain: sleep duration*	±	Very low	-	Very low	±	Very low
Japan Children’s Study Sleep Questionnaire (JCSSQ)^*^ [39]						
* Domain: sleep duration*	±	Very low	-	Very low	-	Very low
* Domain: sleep efficiency*	±	Very low	-	Very low	-	Very low
Japanese Sleep Questionnaire for Elementary Schoolers (JSQ-ES)^**^ [40]						
* Excessive daytime sleepiness*	±	Very low	-	Very low	±	Very low
* Irregular/delayed sleep phase*	±	Very low	±	Very low	±	Very low
**MyDailyMoves (MDM) [41]**	+	Moderate	-	Moderate	+	Moderate
**Pediatric Daytime Sleepiness Scale (PDSS) [42]**	±	Very low	±	Very low	±	Very low
**Pediatric Sleep Practices Questionnaire (PSPQ) [43]**						
* Sleep timing –* * domain: duration*	±	Moderate	±	Moderate	+	Moderate
* Sleep timing –* * domain: timing*	±	Moderate	±	Moderate	+	Moderate
* Sleep routines and consistency*	±	Moderate	±	Moderate	+	Moderate
* Technology use before bedtime*	±	Moderate	±	Moderate	+	Moderate
* Sleep environment*	±	Moderate	±	Moderate	+	Moderate
**Pictorial Sleepiness Scale (PSS) [44]**	+	Very low	-	Very low	+	Very low
**PROMIS Pediatric Sleep Health Items (PROMIS-PSHI) [45]**						
* Sleep onset*	±	Moderate	±	Moderate	+	Moderate
* Sleep continuity*	±	Moderate	±	Moderate	+	Moderate
* Sleep quality*	±	Moderate	±	Moderate	+	Moderate
* Daytime sleepiness*	±	Moderate	±	Moderate	+	Moderate
* Sleep offset*	±	Moderate	±	Moderate	+	Moderate
**“Simple Self-Report Sleep Questionnaire” (SSRSQ) [46]**	±	Very low	-	Very low	±	Very low
**Sleep and Lifestyle Questionnaire (SLQ) [47]**						
* Domain: sleep duration*	±	Very low	-	Very low	±	Very low
* Domain: sleep efficiency*	±	Very low	-	Very low	±	Very low
* Domain: timing*	±	Very low	-	Very low	±	Very low
* Domain: daytime sleepiness*	±	Very low	-	Very low	±	Very low
* Domain: behaviors*	±	Very low	-	Very low	±	Very low
**Sleep Self Report (SSR) [48, 49]**						
* Domain: sleep quality*	±	Very low	-	Very low	±	Very low
* Domain: sleep efficiency*	±	Very low	-	Very low	±	Very low
* Domain: timing*	±	Very low	-	Very low	±	Very low
* Domain: daytime sleepiness*	±	Very low	-	Very low	±	Very low
* Domain: behaviors*	±	Very low	-	Very low	±	Very low
Sleep Timing Questionnaire (STQ) [50, 51]^***^						
* Domain: duration*	±	Very low	±	Very low	±	Very low
* Domain: sleep efficiency*	±	Very low	-	Very low	±	Very low
* Domain: timing*	±	Very low	±	Very low	±	Very low
* Domain: behaviors*	±	Very low	±	Very low	±	Very low

*Abbreviations:* + = satisfactory results; − = unsatisfactory results; ± = inconsistent results; ? = indeterminate

NA = not applicable

* Instrument not available. Only PROM development study was rated

** Reviewers rated the English version

*** Development study of adult sample was evaluated

Results on (aspects of) sufficient content validity are presented in green

For none of the sleep health domains sufficient results on all aspects of content validity were found (Table 3). For measuring *Sleep Duration*, we found sufficient relevance of MDM and sufficient comprehensibility of MDM and the PSPQ subscale Sleep Timing, with moderate quality of evidence. For measuring *Sleep Quality*, we found sufficient relevance of MDM and sufficient comprehensibility of MDM and the PROMIS-PSHI subscale Sleep Quality and the subscale Sleep Offset, all with moderate quality of evidence. For measuring *Sleep Efficiency*, we found sufficient relevance for MDM and sufficient comprehensibility of the subscales Sleep Onset and Sleep Continuity of the PROMIS-PSHI, all with moderate quality of evidence. We also found sufficient comprehensibility for CSWS subscales Falling Asleep and Reinitiating Sleep, with low quality evidence. For measuring *Sleep Timing*, we found sufficient comprehensibility of the subscale Sleep Timing of the PSPQ, with moderate quality of evidence. For measuring *Daytime Sleepiness*, we found sufficient relevance for MDM and the PSS, with moderate and very low quality of evidence, respectively. We also found sufficient comprehensibility of the CSWS subscale Returning to Wakefulness, MDM, the PSS and the PROMIS-PSHI subscale Daytime Sleepiness. Quality of evidence ranged from very low to moderate. For measuring *Sleep related Behaviors,* we found sufficient relevance for MDM, with moderate quality evidence. We found sufficient comprehensibility of the CSWS subscale Going to Bed, MDM, and the PSPQ subscales Sleep Routines and Consistency, Technology Use Before Bedtime, and Sleep Environment. Quality of evidence was moderate, except for the CSWS which was low.

**Table 3.** Instruments and subscales with sufficient aspects of content validity including quality of evidence, per domain of sleep health

	Relevance	Comprehensibility
**Sleep duration**	MyDailyMoves*(moderate)*	MyDailyMoves*(moderate);*Pediatric Sleep Practices Questionnaire, subscale: sleep timing*(moderate)*
**Sleep quality**	MyDailyMoves*(moderate)*	MyDailyMoves*(moderate);*PROMIS Pediatric Sleep Health Items, subscale: sleep quality*(moderate);*PROMIS Pediatric Sleep Health Items, subscale: sleep offset*(moderate)*
**Sleep efficiency**	MyDailyMoves*(moderate)*	PROMIS Pediatric Sleep Health Items, subscale: sleep onset *(moderate);* PROMIS Pediatric Sleep Health Items, subscale: sleep continuity *(moderate);* Children’s Sleep Wake Scale, subscale: falling asleep *(low);* Children’s Sleep Wake Scale, subscale: reinitiating sleep *(low)*
**Timing**		Pediatric Sleep Practices Questionnaire, subscale: sleep timing*(moderate)*
**Daytime sleepiness**	MyDailyMoves *(moderate);* Pictorial Sleepiness Scale *(very low)*	Children’s Sleep Wake Scale, subscale: returning to wakefulness*(low);*MyDailyMoves*(moderate);*Pictorial Sleepiness Scale*(very low)*; PROMIS Pediatric Sleep Health Items, subscale: daytime sleepiness*(moderate)*
**Sleep-related behaviors**	MyDailyMoves*(moderate)*	Children’s Sleep Wake Scale, subscale: going to bed*(low);*MyDailyMoves*(moderate);*Pediatric Sleep Practices Questionnaire, subscale: sleep routines and consistency*(moderate);*Pediatric Sleep Practices Questionnaire, subscale: technology use before bedtime*(moderate);*Pediatric Sleep Practices Questionnaire, subscale: sleep environment*(moderate)*

Quality of evidence: very low – low – moderate - high

Comprehensiveness is not reported due to the lack of studies on this measurement property

- *Reviewer’s rating of instruments*

Details of the reviewer’s ratings of instruments can be found in Appendix 4. Sufficient results were mostly found for instruments’ comprehensibility, followed by sufficient results for relevance and comprehensiveness.

### Internal structure: structural validity, internal consistency, and cross-cultural validity

Structural validity was assessed for two instruments: the Children’s Sleep Wake Scale and the Pediatric Sleep Practices Questionnaire (Table 4). For both instruments confirmatory factor analyses were performed. The results for the Children’s Sleep Wake Scale were indeterminate, with high quality of evidence. The results for the Pediatric Sleep Practices Questionnaire were sufficient, with high quality of evidence. Internal consistency was assessed for one instrument: the Children’s Sleep Wake Scale. Internal consistency was assessed by calculating Cronbach’s alpha for the total scale and each subscale and demonstrated sufficient results. The quality of evidence was high. Cross-cultural validity was not assessed for any of the instruments.

**Table 4.** Structural validity and internal consistency, including methodological quality, results, and quality of evidence of instruments with satisfactory results on aspects of content validity

		Structural validity				Internal consistency			
Instrument	Study population	Methodological quality^a^	Results	Rating of results^b^	Quality of evidence^c^	Methodological quality^a^	Results	Rating of results^b^	Quality of evidence^c^
**Children’s Sleep Wake Scale (CSWS) [36]**	Sample structural validity study: *n* = 751 Age = 6.1 ± 3.1 years (range 2–12) Sex: 50% boys Sample internal consistency study: *n* = 543 Age = 4.9 ± 2.0 years (range 2–8) Sex: 51% boys	Very good	CFA: 5-factor solution with eigenvalues >1.00, accounting for 64.2% of the variance	?	High	Very good	Total scale Cronbach’s α = 0.89. Subscales Cronbach’s α: going to bed (α = 0.88), falling asleep (α = 0.83), maintaining sleep (α = 0.81), reinitiating sleep (α = 0.81), and returning to wakefulness (α = 0.91)	**+**	High
**Pediatric Sleep Practices Questionnaire (PSPQ) [43]**	*n* = 169 Age = unknown (range 8–12 years)	Very good	CFA: Comparative fit index = 1.00, Tucker-Lewis index = 0.99, root mean square error of approximation = 0.04	**+**	High				

^a^Methodological quality based on the COSMIN risk of bias checklist

^b^Rated against criteria of good measurement properties COSMIN guideline (+ = sufficient; - = insufficient;? = indeterminate)

^c^Graded using GRADE approach COSMIN guideline

### Remaining measurement properties: reliability, measurement error, criterion validity, construct validity, and responsiveness

Reliability was assessed for one instrument: the Children’s Sleep Wake Scale (Table 5). For this instrument one month reliability was assessed by calculating correlations for the total scale and subscales. The results were rated as indeterminate, with low quality evidence. Construct validity was assessed for the Children’s Sleep Wake Scale (Table 6) in which the instrument was compared with a sleep diary and actigraphy. The results were sufficient although with very low quality evidence. A construct validity study was also performed for the Pediatric Sleep Practices Questionnaire, however, as no hypotheses were formulated in the study, construct validity could not be assessed. For none of the instruments measurement error, criterion validity and responsiveness was assessed.

**Table 5.** Reliability, including methodological quality, results, and quality of evidence of instruments with satisfactory results on aspects of content validity

	Reliability					
Instrument	Study population	Time interval	Methodological quality^a^	Results	Rating of results^b^	Quality of evidence^c^
**Children’s Sleep Wake Scale (CSWS) [36]**	*n* = 36 Age = 4.4 ± 2.1 years (range 2–8) Gender = 67% boys	1 month	Doubtful	CSWS total (r = 0.85*), going to bed (*r* = 0.84*), falling asleep (*r* = 0.78*), maintaining sleep (*r* = 0.75*), reinitiating sleep (*r* = 0.67*), and returning to wakefulness (*r* = 0.70*)	?	Low

^a^Methodological quality based on the COSMIN risk of bias checklist

^b^Rated against criteria of good measurement properties COSMIN guideline (+ = sufficient; - = insufficient;? = indeterminate)

^c^Graded using GRADE approach COSMIN guideline

*Significant

**Table 6.** Construct validity (convergent validity and/or discriminative validity), quality of evidence, result rating and methodological quality of instruments with satisfactory results on aspects of content validity

Instrument	Study population	Comparison measure	Methodological quality^a^	Results	Rating of results^b^	Quality of evidence^c^
**Children’s Sleep Wake Scale (CSWS) [36]**	Sleep diary: *n* = 83 Age = 2–8 years Actigraphy: *n* = 69 Age = 2-8 years	Sleep diary Actigraph (AW64, nondominant wrist, 60s epoch)	Inadequate	Sleep diary correlations: CSWS total (*r* = 0.66*), going to bed (*r* = 0.59*), falling asleep (*r* = 0.58*), maintaining sleep (r = 0.72*), reinitiating sleep (r = 0.66*), returning to wakefulness (r = 0.60*) Actigraph correlations: actigraph variables and CSWS total scores (r = -0.46* to 0.41*), sleep latency (min) versus falling asleep (r = 0.61*), sleep minutes (%) versus maintaining sleep (r = 0.54*), sleep efficiency (%) versus maintaining sleep (r = 0.49*), wake bouts (#) versus reinitiating sleep (r = -0.38*), mean wake bouts (min) versus reinitiating sleep (r = -0.49*)	+	Very low

^a^Methodological quality based on the COSMIN risk of bias checklist

^b^Rated against criteria of good measurement properties COSMIN guideline (+ = sufficient; - = insufficient;? = indeterminate)

^c^Graded using GRADE approach COSMIN guideline

*Significant

## Discussion

The current study is, to our knowledge, the first to present an overview of all child- or parent-reported instruments that assess one or more domains of sleep health in a general population of children aged 4–12 years and that have been validated to at least some extend. The first step in this review was to comprehensively evaluate the content validity of all included instruments. None of the (subscales of) instruments demonstrated sufficient results regarding all aspects of content validity. Only sufficient results for relevance and comprehensibility were found for some instruments. None of the (subscales of) instruments demonstrated sufficient comprehensiveness. The quality of evidence of the sufficient results ranged from very low to moderate, but was mostly moderate. In addition, most instruments measured one or more domains of sleep health, but none measured the full construct. All other measurement properties were assessed for the five instruments with indications of sufficient content validity. Some demonstrated sufficient structural validity, internal consistency, and construct validity. The quality of evidence ranged from very low to high.

For 17 out of 20 instruments the quality of development was inadequate. Only 9 out of 20 instruments were developed in a sample of children and/or parents, but mostly quantitative methods were used for identifying relevant items for the instrument. Three instruments were developed together with children and/or parents using qualitative methods: MyDailyMoves [41], the Pediatric Sleep Practices Questionnaire (PSPQ) [43] and the PROMIS Pediatric Sleep Health Items (PROMIS-PSHI) [45]. These instruments were developed more recently compared to other included instruments in this review and showed that a more participatory way of development improved the instrument [41, 45]. The MyDailyMoves study showed that involving the target population during the development may actually lead to a different type of instrument, i.e. a timeline format [41]. Embracing this participatory way of designing instruments opens up valuable opportunities to collect insights from children. Also, during the content validity study of the PROMIS Pediatric Sleep Health Items children indicated that they had poor understanding of nearly half of the suggested items [45]. In addition, in the other study that investigated content validity, children often indicated that they need help in calculating their sleep duration and that they had difficulties in understanding what was meant by *getting ready for bed* [47]. Lately, there has been increasing attention for methodology of instrument development. Improved standards for PROM development may partly explain why the development studies of older instruments more often were rated as inadequate quality. High quality content validity studies can overcome the lack of high quality evidence from inadequate development studies but these studies were rarely done in the field of child sleep health.

For none of the sleep health domains there was evidence for sufficient content validity on all aspects (i.e. relevance, comprehensiveness and comprehensibility). For five out of the six sleep health domains (subscales of) instruments with sufficient relevance and comprehensibility were found: sleep duration, sleep quality, sleep efficiency, daytime sleepiness and sleep-related behaviors. These sufficient results concerned subscales of the Children’s Sleep Wake Scale [36], MyDailyMoves [41], subscales of the Pediatric Sleep Practices Questionnaire [43], the Pictorial Sleepiness Scale [44] and subscales of the PROMIS Pediatric Sleep Health Items [45]. The quality of evidence ranged from very low to moderate, but was mostly moderate. Despite these positive results on one or two aspects of content validity, there is not enough evidence that these instruments adequately measure the constructs.

The results suggest that content validity has not been sufficiently recognized as an important measurement property in the development and evaluation of instruments in the field of child sleep health. Often indeterminate ratings for content validity were given based on the instrument development because the target population was not involved. Despite the lack of involvement of the target population, in several cases the reviewers rated (aspects of) the content validity of these instruments as sufficient. This means that we, as researchers, considered the items relevant for the construct of interest, considered that all aspects of the construct were captured in the items or that the wording of the items was appropriate. This may indicate that a poor quality development study does not need to lead to a poor instrument. However, the ratings of the reviewer were considered as only very low quality evidence. To obtain higher quality evidence for content validity, additional content validity studies with involvement of the target population should be performed.

For the five instruments with indications of sufficient content validity, all additional measurement properties were evaluated. For three instruments additional studies were available. Especially the studies regarding the internal structure of instruments were considered sufficient (i.e. structural validity: *n* = 2 and internal consistency: *n* = 1). The studies on reliability (i.e. test-retest reliability *n* = 1) and construct validity (*n* = 1) showed mixed results in terms of methodological quality, results, and quality of evidence. Besides these studies, other important aspects of validity (e.g. cross-cultural validity, criterion validity) and reliability (e.g. measurement error) were not evaluated for these instruments. In addition, none of the studies evaluated responsiveness. Lack of studies on measurement properties is also demonstrated by other reviews on sleep measures [19–21, 23]. It should be noted that with our approach (i.e. only evaluating additional measurement properties in case of indications of sufficient content validity) studies on measurement properties of the other instruments in this review were not assessed. However, evidence for other measurement properties does not guarantee that an instrument has sufficient relevance and comprehensiveness.

Several aspects should be considered regarding the suitability of instruments for measuring sleep health in primary school-aged children. More than half of the instruments were developed for children older than the age of eight and were mostly child-reported. Children as young as eight years old are able to provide reliable, valid, and meaningful answers on health related questions as long as the instrument is tailored to their developmental age [52]. For children younger than eight years old or children with low literacy levels, parent-reported measures or alternatives might be more suitable. Despite very low evidence of sufficient relevance and comprehensibility the Pictorial Sleepiness Scale might be an alternative, basing its measurements of sleepiness on cartoon faces on which children from the age of four can indicate their perceived sleepiness [44]. Another important aspect to consider when developing a child-reported instrument might be the amount of items, considering children’s attention span. Also recalling “an average week” might be challenging for children and most likely even for parents as they have to combine several nights and weeks into one answer. Few studies reported specifically testing such aspects, yet they seem logically vital to designing a valid measurement tool. By involving the target population in both the conceptual understanding as well as in the practicality of filling out an instrument these aspects can be addressed.

Most instruments in the field of child sleep health measure two or more domains of sleep but do not seem to acknowledge the full multidimensionality of sleep health [10, 11]. The MyDailyMoves instrument measures five of the six domains of sleep health, although quite minimal, which questions the comprehensiveness of the items [41]. The original version of the Children’s Report of Sleep Patterns measures sleep duration, sleep quality, sleep efficiency, and can be extended with an additionally developed subscale for measuring daytime sleepiness [31, 32]. However, these instruments showed inconsistent results for content validity and are therefore not recommended. Given these limitations, it is recommended to consider the multidimensionality of sleep health in the development of new instruments.

The included instruments and subscales cover a broad range of topics related to sleep, but a conceptual framework or clear definition of the construct to be measured was often missing. Therefore the content of subscales often differed between instruments even when they aimed to measure the same construct. This has consequences for measurement properties like responsiveness, but also hampers comparative research. By classifying the instruments according to the definitions of Buysse and Meltzer a clear overview is provided on what (subscales of) instruments are currently available for measuring different domains of sleep health. Although these definitions are the first in acknowledging the complexity and multidimensionality of sleep health, not all domains of the definition are specified into detail and therefore leave room for interpretation. For example, we considered regularity to be part of sleep-related behaviors, whereas this could also be classified under sleep timing. Also, some instruments received an insufficient score for comprehensiveness because they were evaluated against the construct as defined by the reviewers. In addition, some instruments aim to measure one construct but when classified according to our definitions it measured multiple constructs. Therefore some subscales might be interpreted as measuring another construct or measuring another number of constructs.

## Recommendations

Several recommendations for future research can be formulated. When developing an instrument, all aspects of content validity (i.e. relevance, comprehensiveness and comprehensibility) should be thoroughly incorporated, specifically by involving the target population in the development. We also recommend performing high quality studies that comprehensively evaluate other measurement properties (i.e. other aspects of validity, reliability and responsiveness). Finally, when developing an instrument to assess sleep health, the multidimensionality of the construct should be taken into account.

## Strengths and limitations

Strong aspects of our review are its systematic and extensive search strategy, its methodological quality in following the Centre for Reviews and Dissemination (CRD) handbook Systematic Reviews, and its application of the COSMIN approach in evaluating the content validity, taking the quality of the included studies and instrument development into account. Therefore, our review provides a comprehensive overview of instruments and their subscales regarding all aspects of content validity. Separately presenting results regarding relevance, comprehensiveness and comprehensibility provides recommendations for future research. This allows readers to make evidence-informed choices regarding content validity when selecting an instrument or subscale(s) to gain insight into sleep health in a general population of primary school-aged children as well as which instrument or subscale(s) best allows measurement of individual sleep health domains.

A limitation of our study is that only instruments with indications of sufficient (aspects of) content validity were further assessed on other measurement properties. By doing so, we slightly deviated from the COSMIN manual which suggests only to exclude instruments with high quality evidence for insufficient content validity. However, as we wanted to provide the field with an overview of instruments that were at least well developed or that evaluated content validity this was in line with our aim. Another limitation is that based on our classification of sleep health domains, some instruments or subscales received an insufficient rating for content validity regarding to our definition, but that does not imply that the instrument does not properly measure a (slightly) different construct of interest. A last limitation is that we were not able to assess some instruments as we could not retrieve the instrument itself (*n* = 1) or we could only assess the English translation but not the original instrument (*n* = 1).

## Conclusion

Several (subscales of) instruments measuring domains of child sleep health showed sufficient relevance and comprehensibility. However, none of the instruments showed sufficient results on all aspects of content validity. Also, no high quality evidence was available with regard to content validity. Of the (subscales of) instruments that showed sufficient relevance and comprehensibility, some sufficient results on additional measurement properties were found. This study also showed that the currently available instruments measure certain domains of child sleep health instead of measuring the full multidimensional construct. To provide the field with instruments that fully, validly and reliable measure sleep health it is recommended to involve the target population in the development of instruments and to perform high quality content validity studies. High quality studies are also required for the evaluation of other measurement properties to further the evidence of existing instruments.

## Supplementary Material

zsac215_suppl_Supplementary_Material

## Data Availability

The data that support the findings of this review are available from the corresponding author upon reasonable request.
