# Supplementary material for: Measuring sleep health in primary school-aged children: A systematic review of instruments and their content validity
Source: Sleep. 2022 Sep 10;45(11):zsac215. doi: 10.1093/sleep/zsac215 (PMC9644118; doi:10.1093/sleep/zsac215)
Supplement: zsac215_suppl_Supplementary_Material [file zsac215_suppl_supplementary_material.docx]

**Measuring sleep health in primary school-aged children: A systematic review of instruments and their content validity**

Maj-Britt M.R. Inhulsen ^1,2^

Maartje M. van Stralen ^1^

Caroline B. Terwee ^3^

Joanne K. Ujcic-Voortman ^2^

Jacob C. Seidell ^1,2^

Vincent Busch ^2^

1. Department of Health Sciences, Faculty of Science, Vrije Universiteit Amsterdam, Amsterdam Public Health Research Institute, Amsterdam, the Netherlands

2. Sarphati Amsterdam, Public Health Service (GGD), City of Amsterdam, Amsterdam, the Netherlands.

3. Department of Epidemiology and Data Science, Amsterdam UMC, Vrije Universiteit Amsterdam, Amsterdam Public Health Research Institute, the Netherlands

Corresponding author:

Maj-Britt M.R. Inhulsen
Department of Health Sciences, Faculty of Science, Vrije Universiteit Amsterdam
De Boelelaan 1085, 1081 HV Amsterdam, the Netherlands
Email: m.m.r.inhulsen@vu.nl

**Appendix 1. Search strategy**

**PubMed search – 26-07-2021**

[((((((("Sleep"[Mesh:noexp] OR sleep*[tiab] OR time in bed[tiab] OR bed time[tiab] OR bedtime[tiab] OR night rest[tiab] OR night awakening*[tiab] OR night waking*[tiab]))) AND ((instrumentation[sh] OR methods[sh] OR Validation Studies[pt] OR Comparative Study[pt] OR "psychometrics"[MeSH] OR psychometr*[tiab] OR clinimetr*[tw] OR clinometr*[tw] OR "outcome assessment (health care)"[MeSH] OR outcome assessment[tiab] OR outcome measure*[tw] OR "observer variation"[MeSH] OR observer variation[tiab] OR "Health Status Indicators"[Mesh] OR "reproducibility of results"[MeSH] OR reproducib*[tiab] OR "discriminant analysis"[MeSH] OR reliab*[tiab] OR unreliab*[tiab] OR valid*[tiab] OR coefficient[tiab] OR homogeneity[tiab] OR homogeneous[tiab] OR "internal consistency"[tiab] OR (cronbach*[tiab] AND (alpha[tiab] OR alphas[tiab])) OR (item[tiab] AND (correlation*[tiab] OR selection*[tiab] OR reduction*[tiab])) OR agreement[tiab] OR precision[tiab] OR imprecision[tiab] OR "precise values"[tiab] OR test-retest[tiab] OR (test[tiab] AND retest[tiab]) OR (reliab*[tiab] AND (test[tiab] OR retest[tiab])) OR stability[tiab] OR interrater[tiab] OR inter-rater[tiab] OR intrarater[tiab] OR intra-rater[tiab] OR intertester[tiab] OR inter-tester[tiab] OR intratester[tiab] OR intra-tester[tiab] OR interobserver[tiab] OR inter-observer[tiab] OR intraobserver[tiab] OR intra-observer[tiab] OR intertechnician[tiab] OR inter-technician[tiab] OR intratechnician[tiab] OR intra-technician[tiab] OR interexaminer[tiab] OR inter-examiner[tiab] OR intraexaminer[tiab] OR intra-examiner[tiab] OR interassay[tiab] OR inter-assay[tiab] OR intraassay[tiab] OR intra-assay[tiab] OR interindividual[tiab] OR inter-individual[tiab] OR intraindividual[tiab] OR intra-individual[tiab] OR interparticipant[tiab] OR inter-participant[tiab] OR intraparticipant[tiab] OR intra-participant[tiab] OR kappa[tiab] OR kappa's[tiab] OR kappas[tiab] OR repeatab*[tiab] OR ((replicab*[tiab] OR repeated[tiab]) AND (measure[tiab] OR measures[tiab] OR findings[tiab] OR result[tiab] OR results[tiab] OR test[tiab] OR tests[tiab])) OR generaliza*[tiab] OR generalisa*[tiab] OR concordance[tiab] OR (intraclass[tiab] AND correlation*[tiab]) OR discriminative[tiab] OR "known group"[tiab] OR factor analysis[tiab] OR factor analyses[tiab] OR dimension*[tiab] OR subscale*[tiab] OR (multitrait[tiab] AND scaling[tiab] AND (analysis[tiab] OR analyses[tiab])) OR item discriminant[tiab] OR **inter** **scale** correlation*[tiab] OR error[tiab] OR errors[tiab] OR "individual variability"[tiab] OR (variability[tiab] AND (analysis[tiab] OR values[tiab])) OR (uncertainty[tiab] AND (measurement[tiab] OR measuring[tiab])) OR "standard error of measurement"[tiab] OR sensitiv*[tiab] OR responsive*[tiab] OR ((minimal[tiab] OR minimally[tiab] OR clinical[tiab] OR clinically[tiab]) AND (important[tiab] OR significant[tiab] OR detectable[tiab]) AND (change[tiab] OR difference[tiab])) OR (small*[tiab] AND (real[tiab] OR detectable[tiab]) AND (change[tiab] OR difference[tiab])) OR meaningful change[tiab] OR "ceiling effect"[tiab] OR "floor effect"[tiab] OR "Item response model"[tiab] OR IRT[tiab] OR Rasch[tiab] OR "Differential item functioning"[tiab] OR DIF[tiab] OR "computer adaptive testing"[tiab] OR "item bank"[tiab] OR "cross-cultural equivalence"[tiab]))) AND (instrumentation[sh] OR methods[sh] OR Validation Studies[pt] OR Comparative Study[pt] OR "psychometrics"[MeSH] OR psychometr*[tiab] OR clinimetr*[tw] OR clinometr*[tw] OR "outcome assessment (health care)"[MeSH] OR outcome assessment[tiab] OR outcome measure*[tw] OR "observer variation"[MeSH] OR observer variation[tiab] OR "Health Status Indicators"[Mesh] OR "reproducibility of results"[MeSH] OR reproducib*[tiab] OR "discriminant analysis"[MeSH] OR reliab*[tiab] OR unreliab*[tiab] OR valid*[tiab] OR coefficient[tiab] OR homogeneity[tiab] OR homogeneous[tiab] OR "internal consistency"[tiab] OR (cronbach*[tiab] AND (alpha[tiab] OR alphas[tiab])) OR (item[tiab] AND (correlation*[tiab] OR selection*[tiab] OR reduction*[tiab])) OR agreement[tiab] OR precision[tiab] OR imprecision[tiab] OR "precise values"[tiab] OR test-retest[tiab] OR (test[tiab] AND retest[tiab]) OR (reliab*[tiab] AND (test[tiab] OR retest[tiab])) OR stability[tiab] OR interrater[tiab] OR inter-rater[tiab] OR intrarater[tiab] OR intra-rater[tiab] OR intertester[tiab] OR inter-tester[tiab] OR intratester[tiab] OR intra-tester[tiab] OR interobserver[tiab] OR inter-observer[tiab] OR intraobserver[tiab] OR intra-observer[tiab] OR intertechnician[tiab] OR inter-technician[tiab] OR intratechnician[tiab] OR intra-technician[tiab] OR interexaminer[tiab] OR inter-examiner[tiab] OR intraexaminer[tiab] OR intra-examiner[tiab] OR interassay[tiab] OR inter-assay[tiab] OR intraassay[tiab] OR intra-assay[tiab] OR interindividual[tiab] OR inter-individual[tiab] OR intraindividual[tiab] OR intra-individual[tiab] OR interparticipant[tiab] OR inter-participant[tiab] OR intraparticipant[tiab] OR intra-participant[tiab] OR kappa[tiab] OR kappa's[tiab] OR kappas[tiab] OR repeatab*[tiab] OR ((replicab*[tiab] OR repeated[tiab]) AND (measure[tiab] OR measures[tiab] OR findings[tiab] OR result[tiab] OR results[tiab] OR test[tiab] OR tests[tiab])) OR generaliza*[tiab] OR generalisa*[tiab] OR concordance[tiab] OR (intraclass[tiab] AND correlation*[tiab]) OR discriminative[tiab] OR "known group"[tiab] OR factor analysis[tiab] OR factor analyses[tiab] OR dimension*[tiab] OR subscale*[tiab] OR (multitrait[tiab] AND scaling[tiab] AND (analysis[tiab] OR analyses[tiab])) OR item discriminant[tiab] OR inter scale correlation*[tiab] OR error[tiab] OR errors[tiab] OR "individual variability"[tiab] OR (variability[tiab] AND (analysis[tiab] OR values[tiab])) OR (uncertainty[tiab] AND (measurement[tiab] OR measuring[tiab])) OR "standard error of measurement"[tiab] OR sensitiv*[tiab] OR responsive*[tiab] OR ((minimal[tiab] OR minimally[tiab] OR clinical[tiab] OR clinically[tiab]) AND (important[tiab] OR significant[tiab] OR detectable[tiab]) AND (change[tiab] OR difference[tiab])) OR (small*[tiab] AND (real[tiab] OR detectable[tiab]) AND (change[tiab] OR difference[tiab])) OR meaningful change[tiab] OR "ceiling effect"[tiab] OR "floor effect"[tiab] OR "Item response model"[tiab] OR IRT[tiab] OR Rasch[tiab] OR "Differential item functioning"[tiab] OR DIF[tiab] OR "computer adaptive testing"[tiab] OR "item bank"[tiab] OR "cross-cultural equivalence"[tiab])) AND ((child*[tw] OR schoolchild*[tw] OR boy[tw] OR boys[tw] OR boyhood[tw] OR girl[tw] OR girls[tw] OR girlhood[tw] OR preschool*[tw]))) AND ((disease*[ti] OR syndrome[tw] OR study protocol[tw] OR apnea[tw] OR epilep*[tw] OR narcolep*[tw] OR tonsil*[tw] OR Sudden Unexpected Death in Infancy[tw] OR SUDI[tw] OR apnoea[tw] OR OSA[tw] OR malaria[tw] OR hiv[tw] OR adhd[tw] OR Attention Deficit Disorder with Hyperactivity[tw]))) NOT ((("addresses"[Publication Type] OR "biography"[Publication Type] OR "case reports"[Publication Type] OR "comment"[Publication Type] OR "directory"[Publication Type] OR "editorial"[Publication Type] OR "festschrift"[Publication Type] OR "interview"[Publication Type] OR "lectures"[Publication Type] OR "legal cases"[Publication Type] OR "legislation"[Publication Type] OR "letter"[Publication Type] OR "news"[Publication Type] OR "newspaper article"[Publication Type] OR "patient education handout"[Publication Type] OR "popular works"[Publication Type] OR "congresses"[Publication Type] OR "consensus development conference"[Publication Type] OR "consensus development conference, nih"[Publication Type] OR "practice guideline"[Publication Type]) NOT (animals[mh] NOT (humans[mh] AND animals[mh]))))](https://www.ncbi.nlm.nih.gov/pubmed?term=(((((((%22Sleep%22%5bMesh:noexp%5d%20OR%20sleep*%5btiab%5d%20OR%20time%20in%20bed%5btiab%5d%20OR%20bed%20time%5btiab%5d%20OR%20bedtime%5btiab%5d%20OR%20night%20rest%5btiab%5d%20OR%20night%20awakening*%5btiab%5d%20OR%20night%20waking*%5btiab%5d)))%20AND%20((instrumentation%5bsh%5d%20OR%20methods%5bsh%5d%20OR%20Validation%20Studies%5bpt%5d%20OR%20Comparative%20Study%5bpt%5d%20OR%20%22psychometrics%22%5bMeSH%5d%20OR%20psychometr*%5btiab%5d%20OR%20clinimetr*%5btw%5d%20OR%20clinometr*%5btw%5d%20OR%20%22outcome%20assessment%20(health%20care)%22%5bMeSH%5d%20OR%20outcome%20assessment%5btiab%5d%20OR%20outcome%20measure*%5btw%5d%20OR%20%22observer%20variation%22%5bMeSH%5d%20OR%20observer%20variation%5btiab%5d%20OR%20%22Health%20Status%20Indicators%22%5bMesh%5d%20OR%20%22reproducibility%20of%20results%22%5bMeSH%5d%20OR%20reproducib*%5btiab%5d%20OR%20%22discriminant%20analysis%22%5bMeSH%5d%20OR%20reliab*%5btiab%5d%20OR%20unreliab*%5btiab%5d%20OR%20valid*%5btiab%5d%20OR%20coefficient%5btiab%5d%20OR%20homogeneity%5btiab%5d%20OR%20homogeneous%5btiab%5d%20OR%20%22internal%20consistency%22%5btiab%5d%20OR%20(cronbach*%5btiab%5d%20AND%20(alpha%5btiab%5d%20OR%20alphas%5btiab%5d))%20OR%20(item%5btiab%5d%20AND%20(correlation*%5btiab%5d%20OR%20selection*%5btiab%5d%20OR%20reduction*%5btiab%5d))%20OR%20agreement%5btiab%5d%20OR%20precision%5btiab%5d%20OR%20imprecision%5btiab%5d%20OR%20%22precise%20values%22%5btiab%5d%20OR%20test-retest%5btiab%5d%20OR%20(test%5btiab%5d%20AND%20retest%5btiab%5d)%20OR%20(reliab*%5btiab%5d%20AND%20(test%5btiab%5d%20OR%20retest%5btiab%5d))%20OR%20stability%5btiab%5d%20OR%20interrater%5btiab%5d%20OR%20inter-rater%5btiab%5d%20OR%20intrarater%5btiab%5d%20OR%20intra-rater%5btiab%5d%20OR%20intertester%5btiab%5d%20OR%20inter-tester%5btiab%5d%20OR%20intratester%5btiab%5d%20OR%20intra-tester%5btiab%5d%20OR%20interobserver%5btiab%5d%20OR%20inter-observer%5btiab%5d%20OR%20intraobserver%5btiab%5d%20OR%20intra-observer%5btiab%5d%20OR%20intertechnician%5btiab%5d%20OR%20inter-technician%5btiab%5d%20OR%20intratechnician)

**PsycInfo – 26-07-2021**

Sleep OR "time in bed" OR "bed time" OR bedtime OR "night rest" OR "night awakening*" OR "night waking*" AND instrumentation OR Validation Studies OR "reproducibility of results" OR reproducib* OR "psychometrics" OR psychometr* OR clinimetr* OR clinometr* OR "observer variation" OR "discriminant analysis" OR reliab* OR valid* OR validity OR coefficient OR "internal consistency" OR cronbach* OR "item correlation" OR "item correlations" OR "item selection" OR "item selections" OR "item reduction" OR "item reductions" OR agreement OR precision OR imprecision OR "precise values" OR interrater OR inter-rater OR intrarater OR intra-rater OR intertester OR inter-tester OR intratester OR intra-tester OR interobserver OR inter-observer OR inter-individual OR intra-observer OR intertechnician OR inter-technician OR intratechnician OR intra-technician OR interexaminer OR intraexaminer OR intra-examiner OR interassay OR intraindividual OR intraparticipant OR interparticipant OR inter-participant OR intra-individual OR interindividual OR intraobserver OR repeatab* OR kappa OR kappas OR "coefficient of variation" OR intra-participant OR "interscale correlation" OR "known group" OR "factor analysis" OR "factor analyses" OR "factor structure" OR "factor structures" OR dimensionality OR subscale* OR "multitrait scaling analysis" OR "multitrait scaling analyses" OR "item discriminant" OR discriminative OR "interscale correlations" OR accuracy OR accurate OR precision OR "variability analysis" OR "interval variability" OR "rate variability" OR "individual variability" OR "meaningful change" OR "cross-cultural equivalence" OR "minimal important difference" OR "minimally important change" OR "minimally important difference" OR "minimal detectable change" OR "minimal detectable difference" OR "minimally detectable change" OR "minimally detectable difference" OR "minimal real change" OR "minimal real difference" OR "minimally real change" OR "minimally real difference" OR "ceiling effect" OR "floor effect" OR "Item response model" OR IRT OR Rasch OR "Differential item functioning" OR "computer adaptive testing" OR "item bank" OR "minimal important change" AND Child OR children OR child* OR "school age" OR preschool* OR boy OR boys OR boyhood OR girl OR girls OR girlhood

**Web of Science – 26-07-2021**

(TS=(sleep* OR "time in bed" OR "bed time" OR bedtime OR "night rest" OR "night awakening*" OR "night waking*")) AND (TS=(instrumentation OR Validation Studies OR "reproducibility of results" OR reproducib* OR "psychometrics" OR psychometr* OR clinimetr* OR clinometr* OR "observer variation" OR "discriminant analysis" OR reliab* OR valid* OR coefficient OR "internal consistency" OR cronbach* OR "item correlation" OR "item correlations" OR "item selection" OR "item selections" OR "item reduction" OR "item reductions" OR agreement OR precision OR imprecision OR "precise values" OR interrater OR inter-rater OR intrarater OR intra-rater OR intertester OR inter-tester OR intratester OR intra-tester OR interobserver OR inter-observer OR inter-individual OR intra-observer OR intertechnician OR inter-technician OR intratechnician OR intra-technician OR interexaminer OR intraexaminer OR intra-examiner OR interassay OR intraindividual OR intraparticipant OR interparticipant OR inter-participant OR intra-individual OR interindividual OR intraobserver OR repeatab* OR kappa OR kappas OR "coefficient of variation" OR intra-participant OR "interscale correlation" OR "known group" OR "factor analysis" OR "factor analyses" OR "factor structure" OR "factor structures" OR dimensionality OR subscale* OR "multitrait scaling analysis" OR "multitrait scaling analyses" OR "item discriminant" OR discriminative OR "interscale correlations" OR accuracy OR accurate OR precision OR "variability analysis" OR "interval variability" OR "rate variability" OR "individual variability" OR "meaningful change" OR "cross-cultural equivalence" OR "minimal important difference" OR "minimally important change" OR "minimally important difference" OR "minimal detectable change" OR "minimal detectable difference" OR "minimally detectable change" OR "minimally detectable difference" OR "minimal real change" OR "minimal real difference" OR "minimally real change" OR "minimally real difference" OR "ceiling effect" OR "floor effect" OR "Item response model" OR IRT OR Rasch OR "Differential item functioning" OR "computer adaptive testing" OR "item bank" OR "minimal important change")) AND (TS=(Child OR children OR child* OR "school age" OR preschool* OR boy OR boys OR boyhood OR girl OR girls OR girlhood) OR TI=(Child OR children OR child* OR "school age" OR preschool* OR boy OR boys OR boyhood OR girl OR girls OR girlhood))

**EmBase – 26-07-2021**

child:ab,ti OR boys:ab,ti OR girl:ab,ti OR boy:ab,ti OR youth:ab,ti OR children:ab,ti OR school:ab,ti AND ('bed time':ab,ti OR sleep:ab,ti OR bedtime:ab,ti OR 'night awakening':ab,ti OR 'night rest':ab,ti OR 'sleepiness':ab,ti OR 'time in bed':ab,ti) AND ( 'intermethod comparison'/exp OR 'data collection method'/exp OR 'validation study'/exp OR 'feasibility study'/exp OR 'pilot study'/exp OR 'psychometry'/exp OR 'reproducibility'/exp OR reproducib*:ab,ti OR 'audit':ab,ti OR psychometr*:ab,ti OR clinimetr*:ab,ti OR clinometr*:ab,ti OR 'observer variation'/exp OR 'observer variation':ab,ti OR 'discriminant analysis'/exp OR 'validity'/exp OR reliab*:ab,ti OR valid*:ab,ti OR 'coefficient':ab,ti OR 'internal consistency':ab,ti OR (cronbach*:ab,ti AND ('alpha':ab,ti OR 'alphas':ab,ti)) OR 'item correlation':ab,ti OR 'item correlations':ab,ti OR 'item selection':ab,ti OR 'item selections':ab,ti OR 'item reduction':ab,ti OR 'item reductions':ab,ti OR 'agreement':ab,ti OR 'precision':ab,ti OR 'imprecision':ab,ti OR 'precise values':ab,ti OR 'test-retest':ab,ti OR ('test':ab,ti AND 'retest':ab,ti) OR (reliab*:ab,ti AND ('test':ab,ti OR 'retest':ab,ti)) OR 'stability':ab,ti OR 'interrater':ab,ti OR 'inter-rater':ab,ti OR 'intrarater':ab,ti OR 'intra-rater':ab,ti OR 'intertester':ab,ti OR 'inter-tester':ab,ti OR 'intratester':ab,ti OR 'intra-tester':ab,ti OR 'interobeserver':ab,ti OR 'inter-observer':ab,ti OR 'intraobserver':ab,ti OR 'intra-observer':ab,ti OR 'intertechnician':ab,ti OR 'inter-technician':ab,ti OR 'intratechnician':ab,ti OR 'intra-technician':ab,ti OR 'interexaminer':ab,ti OR 'inter-examiner':ab,ti OR 'intraexaminer':ab,ti OR 'intra-examiner':ab,ti OR 'interassay':ab,ti OR 'inter-assay':ab,ti OR 'intraassay':ab,ti OR 'intra-assay':ab,ti OR 'interindividual':ab,ti OR 'inter-individual':ab,ti OR 'intraindividual':ab,ti OR 'intra-individual':ab,ti OR 'interparticipant':ab,ti OR 'inter-participant':ab,ti OR 'intraparticipant':ab,ti OR 'intra-participant':ab,ti OR 'kappa':ab,ti OR 'kappas':ab,ti OR 'coefficient of variation':ab,ti OR repeatab*:ab,ti OR (replicab*:ab,ti OR 'repeated':ab,ti AND ('measure':ab,ti OR 'measures':ab,ti OR 'findings':ab,ti OR 'result':ab,ti OR 'results':ab,ti OR 'test':ab,ti OR 'tests':ab,ti)) OR generaliza*:ab,ti OR generalisa*:ab,ti OR 'concordance':ab,ti OR ('intraclass':ab,ti AND correlation*:ab,ti) OR 'discriminative':ab,ti OR 'known group':ab,ti OR 'factor analysis':ab,ti OR 'factor analyses':ab,ti OR 'factor structure':ab,ti OR 'factor structures':ab,ti OR 'dimensionality':ab,ti OR subscale*:ab,ti OR 'multitrait scaling analysis':ab,ti OR 'multitrait scaling analyses':ab,ti OR 'item discriminant':ab,ti OR 'interscale correlation':ab,ti OR 'interscale correlations':ab,ti OR ('error':ab,ti OR 'errors':ab,ti AND (measure*:ab,ti OR correlat*:ab,ti OR evaluat*:ab,ti OR 'accuracy':ab,ti OR 'accurate':ab,ti OR 'precision':ab,ti OR 'mean':ab,ti)) OR 'individual variability':ab,ti OR 'interval variability':ab,ti OR 'rate variability':ab,ti OR 'variability analysis':ab,ti OR ('uncertainty':ab,ti AND ('measurement':ab,ti OR 'measuring':ab,ti)) OR 'standard error of measurement':ab,ti OR sensitiv*:ab,ti OR responsive*:ab,ti OR ('limit':ab,ti AND 'detection':ab,ti) OR 'minimal detectable concentration':ab,ti OR interpretab*:ab,ti OR (small*:ab,ti AND ('real':ab,ti OR 'detectable':ab,ti) AND ('change':ab,ti OR 'difference':ab,ti)) OR 'meaningful change':ab,ti OR 'minimal important change':ab,ti OR 'minimal important difference':ab,ti OR 'minimally important change':ab,ti OR 'minimally important difference':ab,ti OR 'minimal detectable change':ab,ti OR 'minimal detectable difference':ab,ti OR 'minimally detectable change':ab,ti OR 'minimally detectable difference':ab,ti OR 'minimal real change':ab,ti OR 'minimal real difference':ab,ti OR 'minimally real change':ab,ti OR 'minimally real difference':ab,ti OR 'ceiling effect':ab,ti OR 'floor effect':ab,ti OR 'item response model':ab,ti OR 'irt':ab,ti OR 'rasch':ab,ti OR 'differential item functioning':ab,ti OR 'dif':ab,ti OR 'computer adaptive testing':ab,ti OR 'item bank':ab,ti OR 'cross-cultural equivalence':ab,ti) AND [humans]/lim AND [embase]/lim AND ([article]/lim OR [article in press]/lim OR [review]/lim) AND [english]/lim

**Appendix 2. Quality of PROM development**

| **PROM/**  **instrument** | **PROM design** | | | | | | | **Cognitive interview study** | | | **Total quality PROM development study** | |
| --- | --- | --- | --- | --- | --- | --- | --- | --- | --- | --- | --- | --- |
|  | **General design requirements** | | | | | **Concept elicitation** | **Total PROM design** | **General design requirements** | **Comprehen-sibility** | **Comprehen-sivenss** | **Total CI study** |  |
|  | **Clear construct** | **Clear origin of construct** | **Clear target population for which the PROM was developed** | **Clear context of use** | **PROM developed in sample representing the target population** |  |  | **CI study performed in sample representing the target population** |  |  |  |  |
|  | I | D | V | V | I |  | I |  |  |  |  |  |
| Bedtime Routines Questionnaire (BRQ) ^27^ | V | V | V | V | I |  | I |  |  |  |  | I |
| Children’s Report of Sleep Patterns (CRSP) ^28^ | V | V | V | V | I |  | I |  |  |  |  | I |
| Children’s Report of Sleep Patterns – sleepiness scale (CRSP-S) ^29^ | V | D | V | V | I |  | I |  |  |  |  | I |
| Children’s Sleep Behavior Scale (CSBS) ^30^ | V | D | V | V | I |  | I |  |  |  |  | I |
| Children’s Sleep Habits Questionnaire (CSHQ) ^31^ | A | A | V | V | I |  | I |  |  |  |  | I |
| ‘CSHQ-short Japan’ (CSHQ-s) ^32^ | V | V | V | V | I |  | I |  |  |  |  | I |
| Children’s Sleep Wake Scale (CSWS) ^33^ | V | V | V | V | V | D | D | I |  |  |  | I |
| Children's Sleep Assessment Questionnaire (CSAQ) ^34^ | V | V | V | V | I |  | I |  |  |  |  | I |
| Health Behaviour in School-aged Children (HBSC) survey ^35^ * | I | D | I | V | I |  | I |  |  |  |  | I |
| Japan Children’s Study Sleep Questionnaire (JCSSQ) ^36^ * | V | V | V | V | V | D | D | I |  |  |  | I |
| Japanese Sleep Questionnaire for Elementary Schoolers (JSQ-ES) ^37^ ** | D | D | V | V | V | D | D | I |  |  |  | I |
| MyDailyMoves (MDM) ^38^ | V | V | V | V | D |  | D | V | V | D | D | D |
| Pediatric Daytime Sleepiness Scale (PDSS) ^39^ | V | V | V | V | V | D | D | I |  |  |  | I |
| Pediatric Sleep Practices Questionnaire (PSPQ) ^40^ | V | V | V | V | V | V | V | V | V | V | V | V |
| Pictorial Sleepiness Scale ^41^ | V | V | V | D | V | I | I | V | A | D | D | I |
| PROMIS Pediatric Sleep Health Items ^42^ | V | V | V | V | A | A | A | V | V | V | V | A |
| ‘Simple Self-Report Sleep Questionnaire’ (SSRSQ) ^43^ | I | D | V | V | I |  | I |  |  |  |  | I |
| Sleep and Lifestyle Questionnaire ^44^ | V | V | V | V | A | I | I | I |  |  |  | I |
| Sleep Self Report ^45,46^ * | I | I | V | D | I |  | I |  |  |  |  | I |
| Sleep Timing Questionnaire ^47,48^ *** | V | V | I | V | I |  | I |  |  |  |  | I |
| V = very good; A = adequate; D = doubtful; I = inadequate. Empty cells indicate that these aspects were not assessed. | | | | | | | | | | | | |

**Appendix 3. Quality of content validity studies**

| **PROM** | **Content validity** | | | | |
| --- | --- | --- | --- | --- | --- |
|  | **Asking patients** | | | **Asking experts** | |
|  | **Relevance** | **Comprehensiveness** | **Comprehensibility** | **Relevance** | **Comprehensiveness** |
| Pediatric Sleep Practices Questionnaire (PSPQ) ^40^ |  |  | D |  |  |
| PROMIS Pediatric Sleep Health Items ^42^ |  |  | D |  |  |
| V = very good; A = adequate; D = doubtful; I = inadequate. Empty cells indicate that studies on these aspects are not available. | | | | | |

**Appendix 4. Reviewer’s rating on the content validity of measurement instruments measuring domains of sleep health in children aged 4-12 years**

|  | **Content validity** | | | | | |
| --- | --- | --- | --- | --- | --- | --- |
|  | **Relevance** | | **Comprehensiveness** | | **Comprehensibility** | |
| **Instrument (and subscales)** | **Reviewers’ rating** | **Quality of evidence†** | **Reviewers’ rating** | **Quality of evidence†** | **Reviewers’ rating** | **Quality of evidence†** |
| **Bedtime Routines Questionnaire (BRQ) ^27^** | + | very low | + | very low | + | very low |
| **Children’s Report of Sleep Patterns (CRSP) ^28^** |  | | | | | |
| *Sleep patterns –*  *Domain: sleep duration* | ± | very low | + | very low | + | very low |
| *Sleep patterns – Domain: sleep quality* | ± | very low | + | very low | + | very low |
| *Sleep patterns – Domain: sleep efficiency* | ± | very low | + | very low | + | very low |
| *Sleep patterns - Timing* | ± | very low | + | very low | + | very low |
| *Sleep Hygiene Index* | ± | very low | ± | very low | + | very low |
| **Children’s Report of Sleep Patterns – sleepiness scale (CRSP-S**) **^29^** | + | very low | + | very low | + | very low |
| **Children’s Sleep Behavior Scale (CSBS) ^30^** | - | very low | - | very low | + | very low |
| **Children’s Sleep Habits Questionnaire (CSHQ) ^31^** |  | | | | | |
| *Bedtime resistance – Domain: behaviors* | + | very low | - | very low | + | very low |
| *Sleep Onset Delay* | + | very low | - | very low | + | very low |
| *Sleep duration* | + | very low | + | very low | + | very low |
| *Daytime sleepiness* | + | very low | + | very low | + | very low |
| ***‘CSHQ-short Japan’ (CSHQ-s)* ^32^** |  | | | | | |
| *Difficulty with morning waking* | ± | very low | - | very low | ± | very low |
| **Children’s Sleep Wake Scale (CSWS) ^33^** |  | | | | | |
| *Going to bed* | ± | low | - | low | + | low |
| *Falling asleep* | ± | low | - | low | + | low |
| *Reinitiating sleep* | ± | low | - | low | + | low |
| *Returning to wakefulness* | ± | low | - | low | + | low |
| **Children's Sleep Assessment Questionnaire (CSAQ) ^34^** |  | | | | | |
| *Sleep Hygiene* | ± | very low | - | very low | ± | very low |
| *Sleep Quality - duration* | ± | very low | - | very low | ± | very low |
| *Sleep quality - efficiency* | ± | very low | - | very low | ± | very low |
| *Sleep quality –*  *timing* | ± | very low | - | very low | ± | very low |
| *Sleep quality – daytime sleepiness* | ± | very low | - | very low | ± | very low |
| **Health Behaviour in School-aged Children (HBSC) survey* ^35^** |  | | | | | |
| *Sleep subscale – Domain: Sleep duration* | ± | very low | - | very low | + | very low |
| **Japan Children’s Study Sleep Questionnaire (JCSSQ)* ^36^** |  | | | | | |
| *Domain: Sleep duration* | NA | very low | NA | very low | NA | very low |
| *Domain: Sleep efficiency* | NA | very low | NA | very low | NA | very low |
| **Japanese Sleep Questionnaire for Elementary Schoolers (JSQ-ES)** ^37^** |  | | | | | |
| *Excessive daytime sleepiness* | ± | very low | - | very low | ± | very low |
| *Irregular/delayed sleep phase* | ± | very low | + | very low | ± | very low |
| **MyDailyMoves (MDM) ^38^** | + | moderate | - | moderate | + | moderate |
| **Pediatric Daytime Sleepiness Scale (PDSS) ^39^** | + | very low | + | very low | + | very low |
| **Pediatric Sleep Practices Questionnaire (PSPQ) ^40^** |  | | | | | |
| *Sleep timing –*  *Domain: duration* | ± | moderate | - | moderate | + | moderate |
| *Sleep timing –*  *Domain: timing* | ± | moderate | + | moderate | + | moderate |
| *Sleep routines and consistency* | + | moderate | - | moderate | + | moderate |
| *Technology use before bedtime* | + | moderate | - | moderate | + | moderate |
| *Sleep environment* | + | moderate | - | moderate | + | moderate |
| **Pictorial Sleepiness Scale (PSS) ^41^** | ± | very low | ± | very low | + | very low |
| **PROMIS Pediatric Sleep Health Items (PROMIS-PSHI) ^42^** |  | | | | | |
| *Sleep onset* | + | moderate | - | moderate | + | moderate |
| *Sleep continuity* | + | moderate | - | moderate | + | moderate |
| *Sleep quality* | + | moderate | + | moderate | + | moderate |
| *Daytime sleepiness* | + | moderate | + | moderate | + | moderate |
| *Sleep offset* | + | moderate | - | moderate | + | moderate |
| **‘Simple Self-Report Sleep Questionnaire’ (SSRSQ) ^43^** | ± | very low | - | very low | ± | very low |
| **Sleep and Lifestyle Questionnaire (SLQ)**^44^ |  | | | | | |
| *Domain: Duration* | ± | very low | - | very low | ± | very low |
| *Domain: Sleep efficiency* | ± | very low | - | very low | ± | very low |
| *Domain: Timing* | ± | very low | - | very low | ± | very low |
| *Domain: Daytime Sleepiness* | ± | very low | - | very low | ± | very low |
| *Domain: Behaviors* | ± | very low | - | very low | ± | very low |
| **Sleep Self Report (SSR) ^45,46^** |  | | | | | |
| *Domain: Sleep Quality* | ± | very low | - | very low | + | very low |
| *Domain: Sleep Efficiency* | ± | very low | - | very low | + | very low |
| *Domain: Timing* | ± | very low | - | very low | + | very low |
| *Domain: Daytime Sleepiness* | ± | very low | - | very low | + | very low |
| *Domain: Behaviors* | ± | very low | - | very low | + | very low |
| **Sleep Timing Questionnaire (STQ) ^47,48^ ***** |  | | | | | |
| *Domain: Duration* | ± | very low | ± | very low | ± | very low |
| *Domain: Sleep Efficiency* | ± | very low | - | very low | ± | very low |
| *Domain: Timing* | ± | very low | + | very low | ± | very low |
| *Domain: Behaviors* | ± | very low | + | very low | ± | very low |
| Abbreviations: + = satisfactory results; − = unsatisfactory results; ± = inconsistent results; ? = indeterminate  NA = not applicable  * Instrument not available. Only PROM development study was rated  ** Reviewers rated the English version  *** Development study of adult sample was evaluated  † Quality of evidence comprises evidence on development and content validity studies  Satisfactory results are presented in green | | | | | | |
